# Supplementary material for: Carbonic Anhydrase-IX Is a Specific and Sensitive Theragnostic Target for Imaging and Radioimmunotherapy in Metastatic Colorectal Cancer
Source: Gastro Hep Adv. 2025 Dec 26;5(3):100871. doi: 10.1016/j.gastha.2025.100871 (PMC12860988; doi:10.1016/j.gastha.2025.100871)
Supplement: Supplemental Methods [file mmc1.pdf]

Gastro Hep Advances 2026;5:100871

ORIGINAL RESEARCH—BASIC

Carbonic Anhydrase-IX Is a Specific and Sensitive Theragnostic Target for Imaging and Radioimmunotherapy in Metastatic Colorectal Cancer

Dijina Swaroop,[1,2,*] Jai Smith,[1,2,*] Jessica Van Zuykelom,[3,4,†] Asif Noor,[5,†] Robin A. Wagner,[1,2] Tamara Vu,[1,2,6] Sarah Lee,[1,2] Alexander G. Heriot,[6] Benjamin Loveday,[6] Kelly Waldeck,[3,4] Peter D. Roselt,[7] Benjamin Blyth,[3,4] Paul S. Donnelly,[5] and Frédéric Hollande[1,2]

[1]Department of Clinical Pathology, Plasticity, Heterogeneity and Tumour Microenvironment International Research Laboratory - PHANTOM - CNRS and The University of Melbourne, Melbourne, Australia; [2]Collaborative Centre for Genomic Cancer Medicine, Victorian Comprehensive Cancer Centre, Melbourne, Australia; [3]Research Division, Models of Cancer Translational Research Centre, Peter MacCallum Cancer Centre, Victoria, Australia; [4]Sir Peter MacCallum Department of Oncology, The University of Melbourne, Victoria, Australia; [5]School of Chemistry and Bio21 Molecular Science and Biotechnology Institute, University of Melbourne, Parkville, Australia; [6]Department of Surgical Oncology, Peter MacCallum Cancer Centre, Melbourne, Australia; and [7]Department of Radiopharmaceutical Sciences, Cancer Imaging, Peter MacCallum Cancer Centre, Melbourne, Australia

Carbonic Anhydrase-IX as a target for imaging and radioimmunotherapy in metastatic colorectal cancer

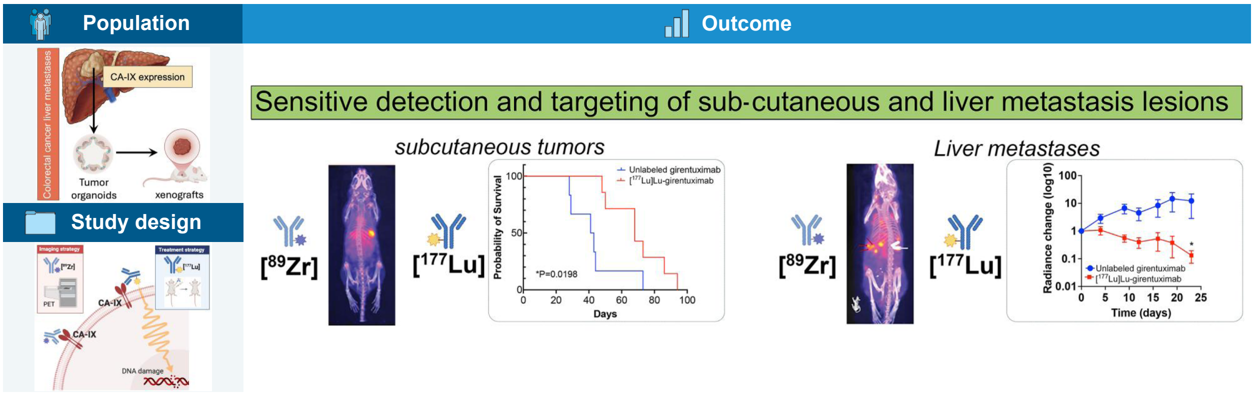

Summary CA-IX is a relevant target for a theragnostic strategy in metastatic colorectal cancer, using radio-labeled girentuximab to image and therapeutically target CA-IX-expressing liver metastases.

Gastro Hep Advances

BACKGROUND AND AIMS: Over 40% of colorectal cancer (CRC) patients develop metastatic disease. Their survival outlook is very low, highlighting the urgent need to improve the detection and therapeutic management of metastatic colorectal cancer (mCRC), particularly when metastases are not surgically resectable. Our study aimed to characterize the preclinical utility of targeting carbonic anhydrase IX (CA-IX) for metastasis imaging and for therapeutic purposes in patients with CRC liver metastases. **METHODS:** CA-IX expression was characterized in 46 liver metastasis samples using RNA sequencing and immunohistochemical staining. We labeled girentuximab, a clinical grade CA-IX antibody, with zirconium-89 ([^{89}Zr]Zr) or lutetium-177 ([^{177}Lu]Lu), and characterized its biodistribution in vivo. Using radiolabeled girentuximab in patient-derived liver metastasis organoids (PDOs) and xenograft models, we then characterized the preclinical utility of CA-IX imaging and therapeutic targeting in mCRC. **RESULTS:** CA-IX mRNA and/or protein expression was detected in 87% of CRC liver metastasis samples, with little to no expression in surrounding liver tissue. Both [^{89}Zr]Zr- and [^{177}Lu]Lu-girentuximab exhibited excellent biodistribution characteristics in mice xenografted with PDOs. Positron emission tomography imaging showed that [^{89}Zr]Zr-girentuximab enabled specific and high-resolution detection of CA-IX-expressing lesions at subcutaneous and hepatic sites compared to [^{18}F]F-fluoro deoxy-glucose. Finally, single-dose [^{177}Lu]Lu-girentuximab treatment induced

*These authors contributed equally to the present work.
†These authors contributed equally to the present work.

Abbreviations used in this paper: **CA-IX, carbonic anhydrase IX; mCRC, metastatic colorectal cancer; PDOs, patient-derived organoids; PET, positron emission tomography.**

Most current article

Copyright © 2025 The Authors. Published by Elsevier Inc. on behalf of the AGA Institute. This is an open access article under the CC BY license (http://creativecommons.org/licenses/by/4.0/).
2772-5723
https://doi.org/10.1016/j.gastha.2025.100871

cytotoxicity in PDOs in vitro and strongly reduced tumor burden in 2 independent xenografted mouse models, with no signs of toxicity. **CONCLUSION:** Our results demonstrate that CA-IX is a relevant target for a theragnostic strategy in mCRC, and provide the first demonstration in clinically-relevant models of metastasis that radiolabeled girentuximab can be used as a scouting agent to stratify and monitor mCRC patients and as a therapeutic alternative for patients with CA-IX-expressing tumors.

Keywords: Bowel Cancer; Theragnostics; Liver Metastases

Introduction

Colorectal cancer (CRC) is the second deadliest of all cancers, and the global burden of the disease is projected to increase up to 68% by 2040.[1] Despite recent advancements in the clinical management of this disease, the 5-year survival rate of CRC patients with distant metastasis remains lower than 15%. The liver is the most prominent site of distant metastasis, with almost 50% of CRC patients manifesting hepatic metastasis during their lifetime, including 25% upon diagnosis.[2] Systemic medical treatment of metastatic colorectal cancer (mCRC) remains largely based on cytotoxic chemotherapy and usually results in poor initial response and/or frequent posttreatment relapse.[3] Few targeted therapies have been adopted in the treatment paradigm for mCRC and they provide small to moderate survival benefit.[3] Immune checkpoint therapy is showing enormous promise in the context of microsatellite instable CRC, but this subtype represents a small minority of cases, leaving the vast majority of mCRC patients with a very poor survival outlook.[3,4] Advancing mCRC treatment and improving imaging modalities to better detect metastases requires specific and sensitive tools and reliable molecular targets with consistent expression in metastases.

Carbonic anhydrase IX (CA-IX) may constitute such a target. CA-IX is a tumor-associated cell surface glycoprotein induced by hypoxia, involved in acidosis adaptation and cancer progression.[5] The restricted physiological expression pattern of CA-IX and its overexpression in cancer tissues make it a potentially attractive clinical target, particularly since it is easily accessible.[6] Elevated expression of CA-IX is also observed in human primary CRC tumors compared to matching normal or normal-adjacent tissues, with higher expression detected in CMS1 and CMS3 molecular subtypes.[7] However, although metastatic dissemination remains the main therapeutic challenge in CRC, the detailed expression pattern of CA-IX and its validity as an imaging and therapeutic target in metastases remains largely unknown.

Girentuximab, a monoclonal antibody that binds to the extracellular proteoglycan-like domain of CA-IX with high specificity and selectivity, may represent an ideal tool to image and target this protein within colorectal liver metastases (LT). There has been a recent surge of interest in the potential of tumor targeted radionuclide therapy where potentially therapeutic β-emitting or α-particle emitting radionuclides are attached to molecules or antibodies that bind selectively to molecular targets enriched in tumor tissue,[8] and girentuximab has yielded promising results for radio-immunoimaging and radio-immunotherapy in metastatic renal cancers.[9,10] Yet, the potential of radiolabeled girentuximab has not been investigated for either diagnostic imaging or radionuclide therapy in robust preclinical models of metastatic CRC.

In this study, we first characterized the expression pattern of CA-IX in liver metastases samples collected from patients with stage IV CRC. Using an innovative desferrioxamine squaramide ethyl ester (H_3DFOSq-OEt)-based approach, we radiolabeled girentuximab with positron-emitting zirconium-89 ([^{89}Zr]Zr) to provide superior imaging characteristics compared to a previous iodine-124-conjugated version of this antibody, including higher tumor uptake, tumor-to-background ratios, and positron emission tomography (PET) resolution.[11,12] In addition, we conjugated 1,4,7,10-tetraazacyclododecane-N,N′,N″,N‴-tetra-acetic acid (DOTA) to girentuximab to allow radiolabeling with lutetium-177 ([^{177}Lu]Lu) for potential therapeutic applications of girentuximab in view of its reported successful delivery of cytotoxic doses to tumors and minimal doses to healthy tissues, resulting in positive treatment responses in advanced renal cell carcinoma.[13–15] We then characterized the potential of [^{89}Zr]Zr or [^{177}Lu]Lu-radiolabeled girentuximab as imaging and therapeutic tools, respectively, using patient-derived liver metastasis organoids (PDOs) and xenografts mice models as preclinical models of metastatic CRC. To our knowledge, this work represents the first preclinical demonstration using patient-derived models that CA-IX is a relevant target in mCRC and that radiolabeled girentuximab can be used to successfully image and target CRC liver metastases.

Materials and Methods

Tumor Sample Collection and Organoid Culture

Primary colorectal tumors and liver metastases (LT) were collected from chemonaive and chemotreated tumors of CRC patients upon surgical tumor resection, as described previously.[16] The use of patient tissues in the study was approved by the Peter MacCallum Cancer Centre Human Research Ethics Committee (HREC/15/PMCC/112, project #15/169) and the Melbourne Health Human Research Ethics Committee (HREC/61352/MH-2020), and written consent was obtained from each patient according to National Health and Medical Research Council guidelines. Liver metastases organoids[16] were grown as described under Supplemental Methods.

Girentuximab Radiolabeling

[^{89}Zr]Zr-girentuximab and [^{177}Lu]Lu-girentuximab were respectively synthesized from H_3DFOSq- and DOTA-girentuximab as described below. The synthesis of DFOSq- and DOTA-girentuximab is detailed in Suppemental Methods.

Synthesis of [^{89}Zr]Zr-girentuximab. [^{89}Zr]Zr^{IV} in 0.05 M oxalic acid (250 μL, 165 MBq, Austin Health) was neutralized with sodium acetate buffer (3 M, 25 μL, final pH 6–7, 0.2 M). A solution of H_3DFOSq-girentuximab (400 μg, 100 μL in 0.9% saline) was added and the reaction mixture was incubated at room temperature for 60 minutes than an aliquot was analyzed and found to have a radiochemical yield of >95%. The crude [^{89}Zr]Zr-girentuximab was purified on a PD-10 size exclusion column (Sephadex G-25, GE healthcare, Cat# 17085101) using 0.5% sodium gentisate in phosphate buffer saline (PBS) (20 mM, pH 7.4) as eluent. The flow through was discarded and multiple fractions (1.0 mL) were collected and 3 fractions with maximum activity were combined to a given 143 MBq (87% isolated radiochemical yeild) in 3 mL with >95% radiochemical purity.

Synthesis of [^{177}Lu]Lu-girentuximab. [^{177}Lu]$LuCl_3$ (900 MBq) in HCl (110 μL, 0.05 M) was neutralized with ammonium acetate solution (1 M, pH 6–7) then DOTA-Girentuximab (1.8 mg) was added. The mixture was heated at 37 °C for 30 minutes. Aliquot was analyzed and found to have a radiochemical yield of >95% with >95% radiochemical purity. The solution was diluted in in PBS (20 mM, pH 7.4, 0.5% sodium gentisate) to make the final doses.

Immunohistochemistry

Tumor samples were fixed in 4% formaldehyde solution, embedded in paraffin, sectioned to 3 μm thickness and stained as previously described[17] and as detailed under Supplemental Methods.

In Vitro *Cytotoxicity Experiments*

pFUGW/Luc2-GFP was transduced into liver metastasis organoids using our previously described protocol.[16] Luciferase-transfected organoids were enzymatically digested into single cells by treating with TrypLE Express for 15–20 minutes. Organoids were plated in black wall 24-well culture plates in Matrigel, supplemented with DMEM-F12 based medium, and exposed to increasing doses of [^{177}Lu]Lu-girentuximab (0, 5, and 10 MBq) or a 100x excess of unlabeled girentuximab for 2 hours, at which point the lutetium containing media was gently removed and growth medium refreshed. Organoid viability was longitudinally monitored by measuring bioluminescence at Day 0, 3, 5, 7, and 9 of [^{177}Lu]Lu-girentuximab exposure. At end point, organoids were incubated with 150 μg/mL D-luciferin diluted in culture medium for 30 minutes. Following removal of the luciferin containing media, luminescence intensity was measured on a BioTek Cytation 3 plate reader (Agilent technologies), and data was represented as % of the growth in unlabeled girentuximab controls.

Animal Experiments

Animal experiments were performed as previously described,[18] under approval from the Peter MacCallum animal ethics Committee (agreements E562 and E654) and in accordance with the Australian code for the care and use of animals for scientific purposes.

Three-month-old female BALB/c nu/nu (BALB/c nude) mice were engrafted with liver metastasis-derived organoids via subcutaneous or intrasplenic inoculation as previously described.[18] Organoids were amplified for injection until reaching the equivalent of 8 confluent wells/mouse in 24-well plates, then Matrigel was dissolved in recovery medium, organoids were pooled and resuspended in a 1:1 Matrigel:PBS mix at the required volume. Inoculation/engraftment procedures are detailed in Supplemental Methods.

Positron Emission Tomography Imaging With [^{89}Zr]Zr-Girentuximab

Xenograft bearing mice received a tail vein injection of ~2.5–3.7 MBq (100–200 μL) of [^{89}Zr]Zr- girentuximab prepared using the above procedures. A 10-minute PET scan, followed by a 1.5 minutes computed tomography, was acquired at 24, 48, 144, and 168 hours postadministration using the Sofie BioSciences Small Animal G8 PET/computed tomography (Perkin Elmer). A region of interest was manually selected, and quantification of tumor uptake is described by SUV_{max} (maximum standardized uptake value, the ratio of the concentration of radioactivity in the highest voxel within the tumor region and the concentration of the radioactivity in the animal). As a clinically-relevant comparison, mice were imaged 1 hour after injection with [^{18}F]F-fluorodeoxyglucose (FDG), since most CRC patients undergo FDG-PET imaging as part of their standard of care management.

In Vivo *Dual Isotope Biodistribution Study*

Animals were randomized and coinjected intravenously with 100 μL of a 1:1 solution of [^{89}Zr]Zr- and [^{177}Lu]Lu-girentuximab prepared using the above procedures. Syringe activity was calibrated pre- and postinjection using the [^{89}Zr]Zr calibration factor, decay corrected and adjusted by a ratio of expected activity:measured activity to determine an injected dose. Injected activities (~4.0–6.0 MBq total) between the 2 isotopes were 1:1 (~2.0–3.0 MBq of each radiotracer per syringe). 24, 48, and 144 hours postinjection (n = 5 mice for each time point), mice were euthanized, and tissues excised, weighed, and counted using a Captus 4000e well counter (Capintec). Each tissue was counted using windowing to determine counts for each isotope, 475–547 KeV for [^{89}Zr]Zr and 187–282 KeV for [^{177}Lu]Lu. The results were decay corrected to the correct isotope and are presented as the percentage injected activity per gram of tissue (% IA/g).

In Vivo *Treatment Using [^{177}Lu]Lu-Girentuximab*

P002_LT organoids were injected subcutaneously into the right flanks of BALB/c nude mice with a 1:1 mixture of Matrigel and PBES in a final volume of 50 μL. Mice were weighed and tumor growth was monitored twice weekly using electronic calipers until tumor volume (0.5 × length × width^2) reached 60–120 mm^3, upon which 5–8 mice/group were randomly assigned to each of 3 groups, respectively, receiving a single injection of saline (0.9% NaCl), 7.4 MBq [^{177}Lu]Lu-girentuximab (2 μ/MBq) or 15 μg unlabeled girentuximab. Tumors were further measured twice weekly until reaching the ethical end point (1200 mm^3).

Results

CA-IX *Expression Pattern in Metastatic Colorectal Cancer Samples and Preclinical Models*

To quantify and characterize the expression pattern of CA-IX in mCRC, CA-IX was first detected using immunohistochemistry in liver metastasis samples collected via surgical resection from 46 patients with stage IV CRC (Figure 1). Scoring and relative

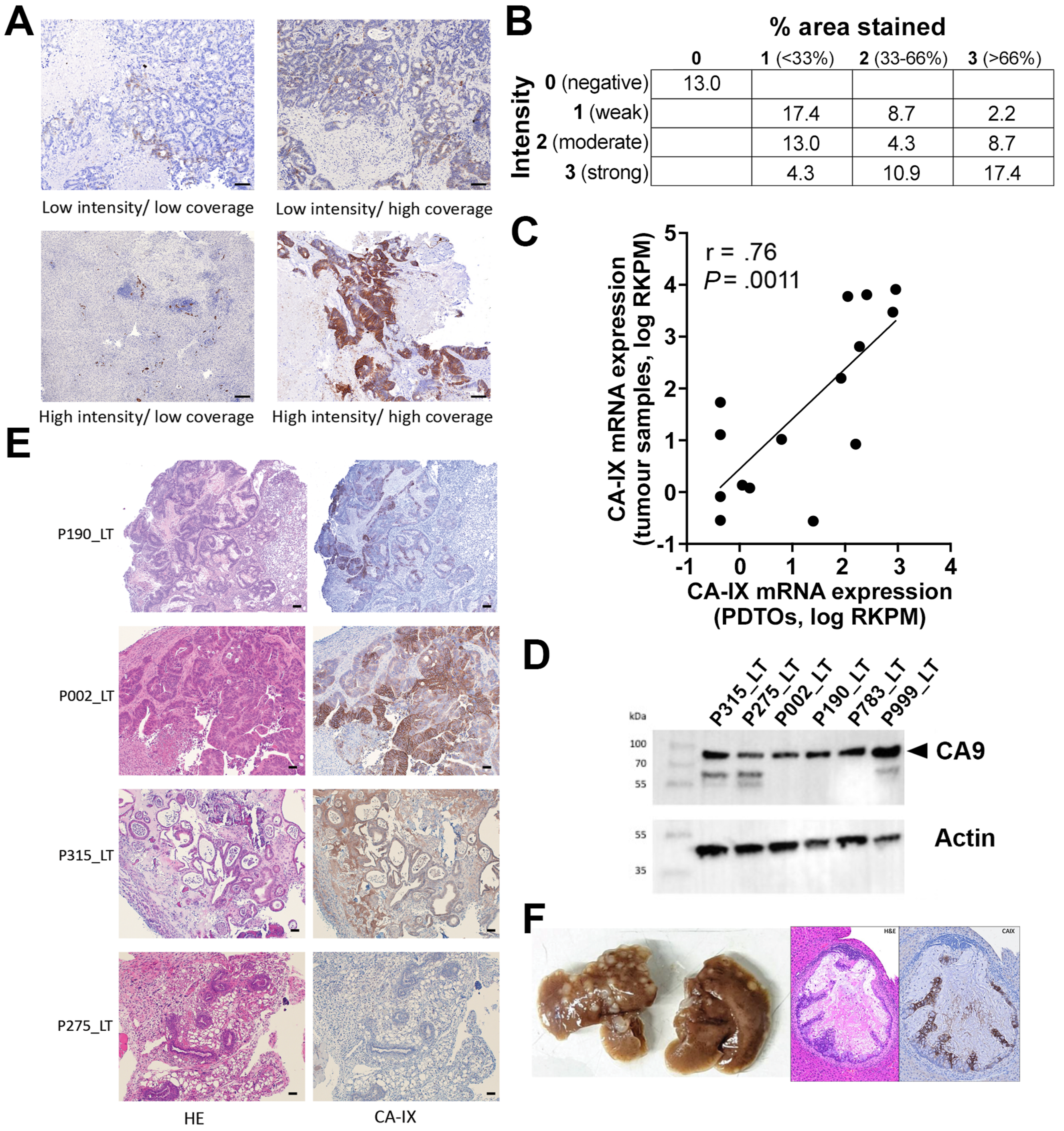

Figure 1. (A) Representative microphotographs of CA-IX immunostaining profiles observed in colorectal cancer (CRC) liver metastasis tissue sections (n = 46). Scale bars = 50 μm. (B) Percentage of liver metastasis samples expressing CA-IX, quantified according to staining intensity (negative to strong) and to the percentage of CA-IX-positive tissue area (0% to >66%). (C) Correlation between CA-IX mRNA expression in CRC liver metastasis tissue samples and matching patient-derived organoids (Spearman correlation, R = 0.70, *P* = .0025, n = 15 patients). (D) Western blot detection of CA-IX in 6 patient-derived liver metastasis organoids (actin, housekeeping control). (E) Representative CA-IX immunostaining in formalin-fixed, paraffin-embedded tissue sections from 4 CRC metastasis organoid-derived xenografts in BALBc/nude mice. Scale bars = 50 μm. (F) Macroscopic tumor detection and representative example of hematoxylin/eosin staining and CA-IX immunostaining in liver metastases generated via intrasplenic inoculation of P190_LT organoids in BALB/c nude mice.

quantification was performed by stratifying samples according to the staining intensity (scored 0–3 for negative, weak, moderate or strong staining, respectively) and to the proportion of tissue area exhibiting any positive staining (0 to 3 for no staining, less than 33%, 33%–66%, or more than 66% of tumor cells stained across the total tissue surface area) (Figure 1A and B, Supplemental Table 1). Most patient samples (87%) demonstrated some degree of CA-IX immunoreactivity, including 41.3% exhibiting moderate to high CA-IX staining across more than 1/3rd of total tissue surface areas (Figure 1A). CA-IX staining was most frequently localized to the cell membrane as expected, although cytoplasmic staining was also detectable in some samples (Figure A1A). Of note, we found very low level of endogenous CA-IX expression in the liver of most patients, enabling clear identification of tumor regions expressing low or high levels of this protein in immunohistochemistry sections (Figure 1A).

Quantification of CA-IX mRNA expression was performed using RNA sequencing data previously generated from 45 liver metastasis tissue samples.[19] The pattern of CA-IX mRNA expression was similar in samples collected prior to (chemo-naive) or after neoadjuvant chemotherapy (Figure A1B). Twenty-one of these samples formed part of the cohort analyzed using immunohistochemistry above, highlighting the significant correlation between CA-IX RNA and protein expression in matching metastatic tumors samples (Spearman correlation, $R = 0.58$, $P = .0033$, $n = 21$) (Figure A1C). We also quantified CA-IX expression at RNA and protein level in PDOs generated from liver metastases described above. RNAseq analysis enabled the robust detection of CAI-IX mRNA in PDOs (Figure A1D) and highlighted a significant positive correlation between CA-IX RNA expression in tumor tissues and matching PDOs (Spearman, $R = 0.70$, $P = .0025$, $n = 15$) (Figure 1C). Immunodetection of CAIX protein expression in PDOs using Western blotting confirmed that tested PDOs expressed CA-IX (Figure 1D); thus representing relevant models for further studies. As previously reported with this polyclonal CA-IX antibody,[20] an additional nonspecific (identified as beta-tubulin) was detected in some but not all organoids (Figure 1D). Heterogeneity of CA-IX expression was recapitulated in vivo within tumors collected from BALB/c nude mice following subcutaneous PDO xenografting (Figure 1E). Liver metastases induced by intrasplenic PDO inoculation also expressed CA-IX (Figure 1F), with enriched expression in hypoxic (pimonidazole-positive) areas (Figure A1E), in agreement with the previously demonstrated link between CA-IX and hypoxia.[21,22]

Taken together, these results suggest that CA-IX is robustly expressed by a large subset of CRC liver metastases, and that in vitro and xenografted PDOs derived from liver metastases represent a reliable model to assess the validity of CA-IX as target for imaging and therapy of mCRC.

Girentuximab Labeling Chemistry

To assess the utility of girentuximab as an imaging and therapeutic tool in mCRC, the antibody was radiolabeled using zirconium-89 and lutetium-177 radionuclides. Girentuximab was conjugated to either H_3DFOSq-OEt or DOTA chelator to prepare H_3DFOSq-girentuximab or DOTA-girentuximab, respectively, with an average chelator to antibody ratio of 3.8–4.2. H_3DFOSq-girentuximab was radiolabeled with $[^{89}Zr]Zr^{IV}$ in sodium acetate buffer at a ratio of 2.5 μg/MBq at room temperature and the progress of the reaction was monitored by size exclusion chromatography. The radio thin layer chromatography (radio-TLC) traces of the reaction mixture after 60 minutes of incubation showed >95% radiochemical yield. Purification using prepacked disposable-10 size exclusion column provided $[^{89}Zr]$Zr-girentuximab in 87% radiochemical yield and >95% radiochemical purity (400 MBq/mg). $[^{177}Lu]$Lu-girentuximab was prepared using DOTA-girentuximab by reacting it with $[^{177}Lu]LuCl_3$ (n.c.a.) in ammonium acetate buffer at a ratio of 2 μg/MBq at 37 °C for 30 minutes. The reaction was monitored by size exclusion chromatography which shows >95% radiochemical yield and radiochemical purity (500 MBq/mg) with no evidence of aggregation. Separate doses for imaging, efficacy and in vitro studies were prepared in PBS (20 mM, pH 7.4, 0.5% gentisate) while 100 MBq of each of the doses were combined for in vivo dual isotope biodistribution study.

Binding Specificity and In Vivo *Biodistribution of* $[^{177}Lu]$*Lu- and* $[^{89}Zr]$*Zr-Labeled Girentuximab*

To investigate the dual biodistribution of $[^{177}Lu]$Lu- and $[^{89}Zr]$Zr-radiolabeled girentuximab, BALB/c nude mice were subcutaneously inoculated with CA-IX-expressing liver metastatic organoids generated from 2 independent patients (yP190 and P002), then concomitantly administered with $[^{177}Lu]$Lu- and $[^{89}Zr]$Zr-girentuximab once tumors were measurable, with or without injection of unlabeled girentuximab. After 24, 48, 144, and/or 168 hours, the cohorts ($n = 3–5$ mice per time point and condition) were euthanized, and the tumor and selected organs were harvested and quantified for radioactivity, using a Captus 4000 well counter (Capintec). A multichannel analyzer was used to enable dual quantification by windowing around the energies of specific gamma emissions from each isotope. Windows of 475–547 KeV (channel 240–275) and 187–282 KeV (channel 98–121) were used respectively for $[^{89}Zr]$Zr and $[^{177}Lu]$Lu. Tumor grafts demonstrated a highly enriched uptake of both $[^{89}Zr]$Zr- and $[^{177}Lu]$Lu-radiolabeled girentuximab over time, compared to nontumor tissues (Figure 2A–D). The tumor uptake of $[^{89}Zr]$Zr-girentuximab was 14.2 ± 2.09 and 21.0 ± 7.36 percent injected dose/gram of tissue (%ID/g) for P002_LT- and yP190_LT-derived tumors, respectively, 24 hours after injection. $[^{177}Lu]$Lu-girentuximab tumor uptake at 24 hours was similar or slightly higher (29.2 ± 5.45 and 21.0 ± 8.72 %ID/g, respectively, for P002_LT- and yP190_LT). Progressive accumulation of radiolabeled girentuximab resulted in higher uptake 144 hours after injection (21.3 ± 3.18 %ID/g for $[^{89}Zr]$Zr- and 33.1 ± 5.21 for

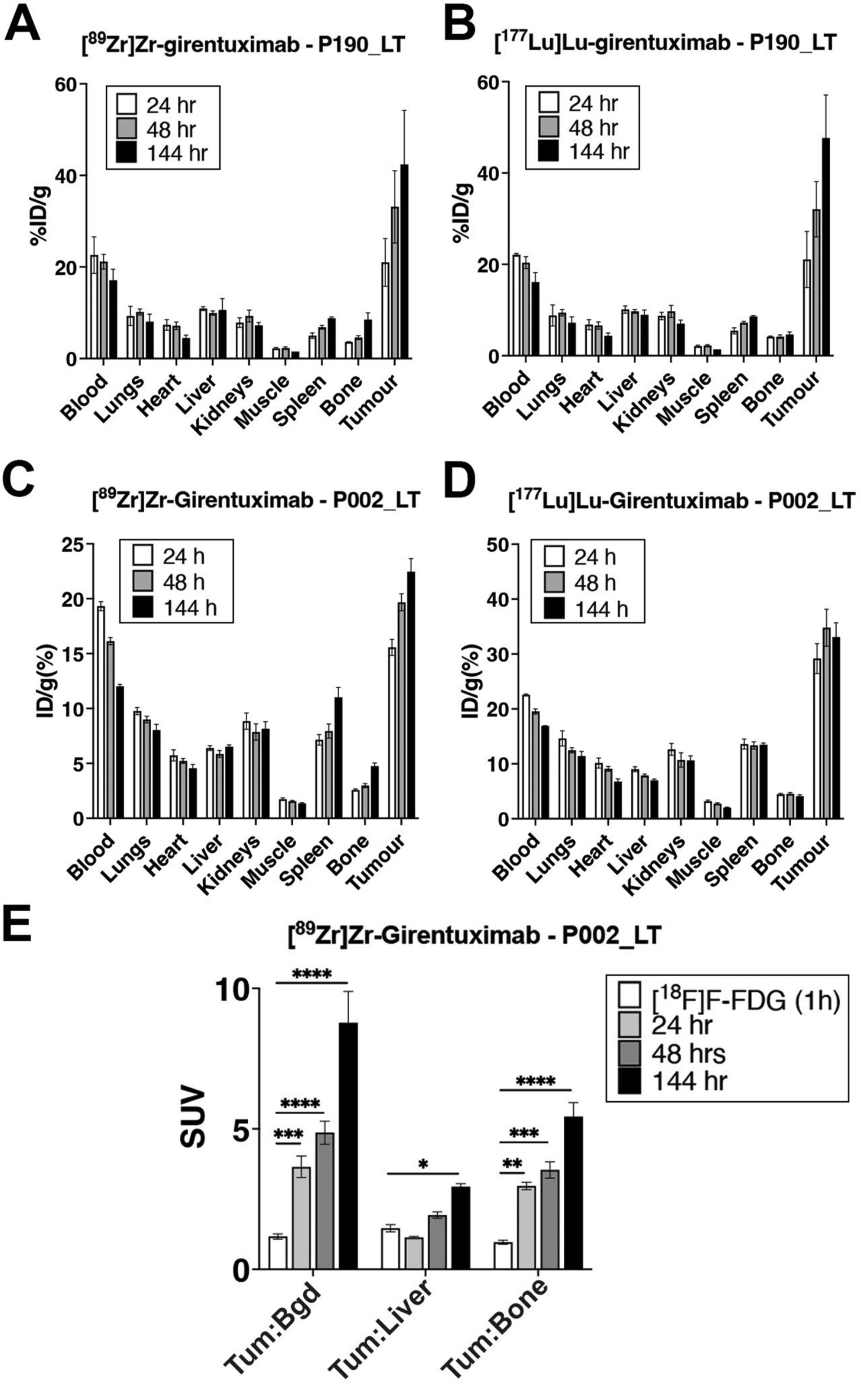

Figure 2. (A and B) Biodistribution of [^{89}Zr]Zr- (A) and [^{177}Lu]Lu-girentuximab (B) 24, 48, and 168 hours after injection into BALBc/nude mice carrying P190_LT-derived xenografts, expressed as mean $\pm$ 1 SEM of %ID/g in collected tumors and organs (n = 3 mice/group). (C and D) Biodistribution of [^{89}Zr]Zr- (C) and [^{177}Lu]Lu-girentuximab (D) 24, 48, 144, and 168 hours after injection into BALBc/nude mice carrying P002_LT-derived xenografts, expressed as mean $\pm$ 1 SEM of %ID/g in collected tumors and organs (n = 5 mice/group). (E) [^{89}Zr]Zr-girentuximab uptake in BALBc/nude mice carrying P002_LT xenografts. Uptake is expressed as mean $\pm$ 1 standard deviation of standardized uptake value ratios above background (Tum:Bgd), liver (Tum:Liver) and bone (Tum:Bone) uptake 24, 48, and 144 hours after injection (n = 5). In comparison, [^{18}F]F-FDG was quantified 1 hour after injection as per routine clinical practice. *, $P < .05$, **$P < .005$, ***$P < .001$, ****$P < .0001$, two-way analysis of variance with Bonferroni's multiple comparison test, n = 5 mice per group.

[^{177}Lu]Lu-girentuximab in P002_LT-derived tumors, and 42.4 $\pm$ 16.7 and 43.6 $\pm$ 14.8 in yP190_LT tumors, respectively). Uptake in nontarget tissue was assessed in blood, lungs, heart, liver, kidneys, muscle, spleen, and bone, and showed a typical biodistribution of a radiolabeled monoclonal antibody with slow blood clearance and excretion via the spleen, liver, and kidneys. %ID/g generally trended down over time and was consistent between [^{177}Lu]Lu- and [^{89}Zr]Zr-radiolabeled girentuximab. Exceptions to this were spleen, which showed static or increased

uptake overtime, and bone, which showed an increase in uptake overtime in both isotopes, but higher [^{89}Zr]Zr-girentuximab accumulation overall (Table A2). This was expected as the spleen is known to act as an antibody sink and accumulate monoclonal antibodies over time, and it is well documented that the bone accumulates unbound [^{89}Zr]Zr, which becomes more available over time as it dissociates from the girentuximab due to radiolysis.[23]

[^{89}Zr]Zr-Girentuximab PET vs [^{18}F]F-FDG-PET Imaging in Metastatic CRC

To characterize the potential of [^{89}Zr]Zr-girentuximab as a radio imaging tracer in CRC liver metastases, PET scanning was performed in BALB/c nude mice following xenografting of liver metastasis PDOs. We first performed subcutaneous inoculation of P002_LT and yP190_LT organoids and, once resulting tumors reached 80–180 mm^3, injected mice with [^{89}Zr]Zr-girentuximab and performed PET imaging 24, 48, 144, and/or 168 hours afterward. We measured selective [^{89}Zr]Zr-girentuximab accumulation in xenografted P002_LT tumors over time, starting from 24 hours (SUVmax = 2.79 ± 0.23; tumor/liver = 1.13 ± 0.06; tumor/bone = 2.97 ± 0.26) and increasing until 144 hours postinjection (SUVmax = 5.44 ± 0.87; tumor/liver = 2.64 ± 0.42; tumor/bone = 5.44 ± 0.99) (Figures 2E and 3A and Table A3). As a comparison, we also imaged mice 1 hours after injection with [^{18}F]F-FDG, which is routinely used in the clinical management of patients with metastatic CRC. Mice injected with [^{18}F]F-FDG did not exhibit any significant tumor-specific accumulation (tumor SUVmax = 0.55 ± 0.08; tumor/liver = 1.46 ± 0.25; tumor/bone = 0.96 ± 0.14) (Figures 2E and 3A). Similar results were obtained with mice inoculated with yP190_LT tumors (Table A3).

To more specifically assess whether girentuximab-based imaging is a promising approach for patients with liver metastases, PET scanning was then performed in BALB/c nude mice following intrasplenic inoculation with Luciferase2-expressing yP190_LT organoids and injection with [^{89}Zr]Zr-girentuximab, in comparison with a standard PET regimen ([^{18}F]F-FDG PET) (Figure 3C and D). PET detection of [^{89}Zr]Zr-girentuximab was performed once liver metastases had successfully implanted, as monitored using bioluminescence imaging (Figure 3C). [^{89}Zr]Zr-girentuximab signal was detected in all mice, while [^{18}F]F-FDG-PET signals were virtually indetectable in mice exhibiting bioluminescent imaging (BLI)-positive liver metastases generated using the same organoid line (Figure 3C). In addition, [^{89}Zr]Zr-girentuximab-based imaging enabled accurate visualization of individual hepatic lesions in the mice, allowing a clear distinction of small tumor lesions located in close proximity to each other (Figure 3D).

Altogether, these experiments demonstrate that [^{89}Zr]Zr-girentuximab is a highly sensitive and specific imaging reagent enabling detection of CA-IX-expressing liver metastatic lesions with high resolution in a preclinical mouse model, even when these tumors are undetectable using classical [^{18}F]F-FDG PET imaging. In addition, we found that [^{89}Zr]Zr-girentuximab displayed lower nonspecific absorption in the brain and urinary bladder when compared to [^{18}F]F-FDG PET (Figure 3A and C), suggesting that [^{89}Zr]Zr-girentuximab is a promising imaging agent in this clinical setting.

Therapeutic Efficacy of [^{177}Lu]Lu-Girentuximab in Preclinical Models

We then radiolabeled girentuximab using [^{117}Lu]Lu, a medium-energy β-emitter and low-energy γ-emitter commonly used as a therapeutic radionuclide,[24] in order to assess the usefulness of this compound for the treatment of CA-IX-expressing liver metastases. We first treated 6 luciferase2-expressing PDOs (P190_LT, P002_LT, P315_LT, P275_LT, P999_LT and P783_LT) with a single dose of 5 or 10 MBq [^{177}Lu]Lu-girentuximab, then quantified their survival using a CTG luciferase reporter assay (Figure 4). We found that 2 organoids (P002_LT and P190_LT, Figure 4A and B) exhibited very high sensitivity to the [^{177}Lu]Lu-girentuximab treatment, with luciferase signals (reflecting organoid numbers) dropping below 10% of those detected in control organoids treated with unlabeled girentuximab from 7 days after treatment. P783_LT displayed transient sensitivity to a single dose of [^{177}Lu]Lu-girentuximab, with 50% and 65% inhibition compared to controls at days 5 and 7, but no significant difference with controls at day 10 (Figure 4C). In contrast, the 3 other lines (P315_LT, P275_LT, P999_LT) did not display sensitivity to [^{177}Lu]Lu-girentuximab under these conditions (Figure 4D–F). Sensitivity of these organoids to [^{177}Lu]Lu-girentuximab treatment was confined to organoids expressing WT TP53 (P190_LT) or carrying a single nucleotide variant of this gene (P002_LT), while those carrying frameshift TP53 variants (P783_LT, P315_LT, P275_LT, P999_LT) exhibited little to no sensitivity to a single dose [^{177}Lu]Lu-girentuximab exposure in vitro (Table A4).

To establish whether [^{177}Lu]Lu-girentuximab treatment can be effective in a preclinical in vivo setting, we investigated its effect in 2 independent mouse models engrafted with CA-IX-expressing organoids, respectively subcutaneously (Figure 5A and B) and in the liver (Figure 5C and D). First, we transplanted P002_LT organoids subcutaneously to BALB/c nude animals and randomly allocated animals to the untreated (0.9% NaCl) control group or to groups receiving respectively a single dose of unlabeled girentuximab (15 μg) or [^{177}Lu]Lu-girentuximab (7.4 MBq) when tumors reached 100 mm^3 (n = 7 mice/group). Tumors of mice treated with vehicle or unlabeled girentuximab grew steadily, with no growth inhibition seen with the unlabeled antibody vs vehicle (repeated measures unpaired T-test, Bonferroni corrected, $P > .6896$). In contrast, the growth of tumors treated with a single dose of [^{177}Lu]Lu-girentuximab was severely impaired (repeated measures unpaired T-test, Bonferroni corrected, $P < .02$ from Day 9–Day 22) vs both control groups, with tumor volume remaining static until day 22 after treatment, after which tumor growth resumed at a slower rate than the

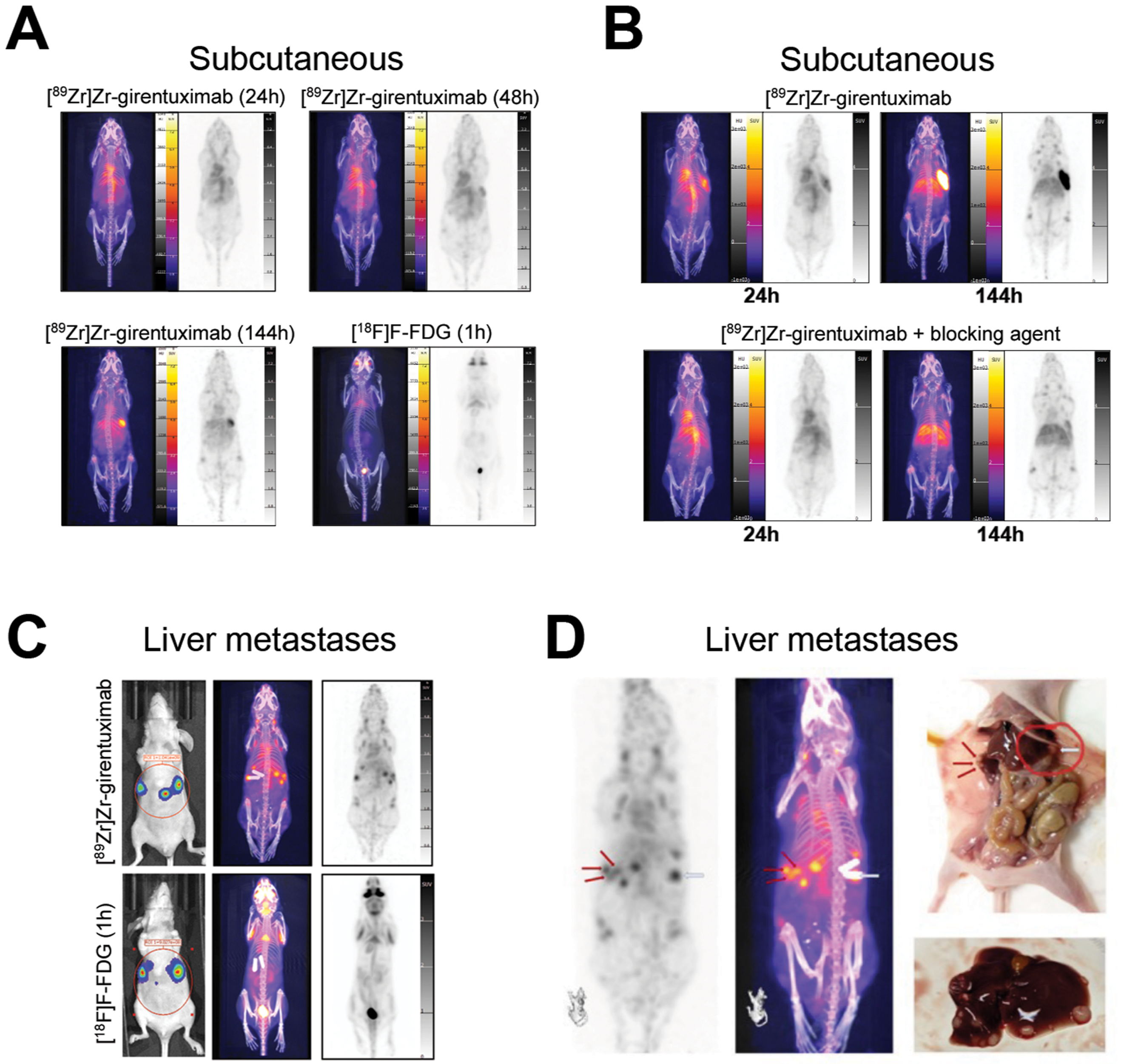

Figure 3. (A) Representative micro-positron emission tomography (microPET) maximum intensity projection (MIP) and computed tomography (CT) images of mice with subcutaneous P002_LT organoid xenografts 24, 48, and 144 hours after injection of [^{89}Zr]Zr-girentuximab or 1 hour after injection of [^{18}F]F-fluoro deoxy-glucose (FDG). (B) MIP and CT images of mice with subcutaneous P002_LT organoid xenografts 24 and 144 hours after injection of [^{89}Zr]Zr-girentuximab in the presence or not of excess unlabeled girentuximab (blocking agent). (C) Bioluminescence imaging (left panels), MIP and CT images of mice bearing liver metastases, 144 hours after injection of [^{89}Zr]Zr-girentuximab or 1 hour after induction of [^{18}F]F-FDG. (D) Representative CT, MIP, and macroscopic autopsy images of multiple liver metastases in a mouse inoculated with P190_LT organoids, 144 hours after injection with [^{89}Zr]Zr-girentuximab. Arrows point to matching metastases detected in different images.

untreated and unlabeled girentuximab groups (Figure 5A). The survival curve showed a significant survival difference (P = .0198) between the group treated with unlabeled girentuximab and [^{177}Lu]Lu-girentuximab (Figure 5B), with the median survival increasing from 42 days to 68 days.

To determine whether [^{177}Lu]Lu-girentuximab can also provide therapeutic benefit in the context of liver metastatic disease, mice intrasplenically inoculated with luciferase2-expressing P190_LT organoids were again treated with a single injection of [^{177}Lu]Lu-girentuximab (7.4 MBq) once tumors were detectable using BLI. In this model, we detected a very strong decrease in the metastatic burden in vivo compared to the burden at treatment onset, whereas control tumors continued to grow during that

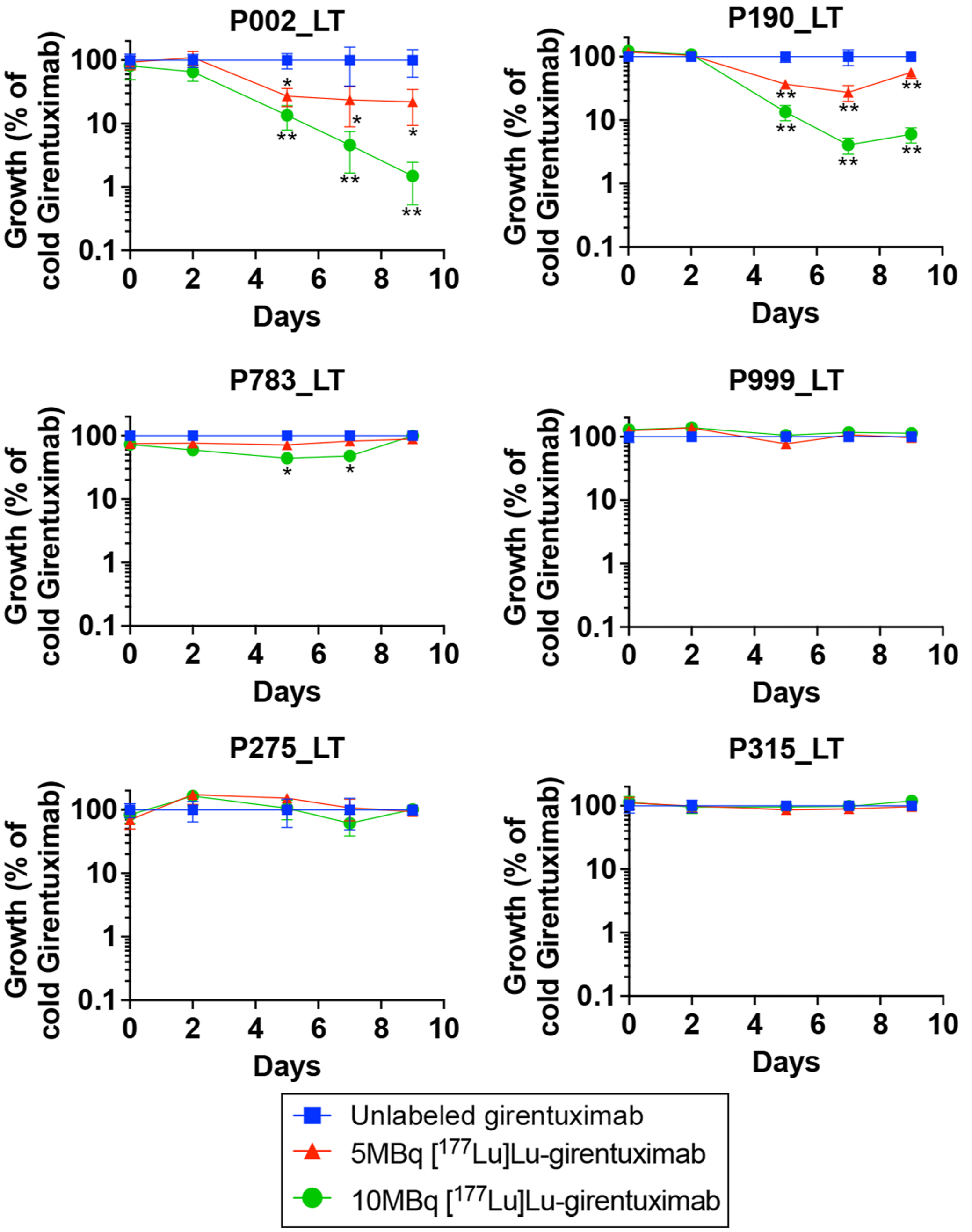

Figure 4. Time course of metastasis organoids survival over 9 days, following single dose treatment with unlabeled girentuximab (blue squares) or to 5 MBq (red triangles) or 10 MBq (green dots) [^{177}Lu]Lu-girentuximab. Results for liver metastases (LT) organoids derived from patients P002, P190, P783, P999, P275, and P315 are expressed as mean ± 1 SEM survival, as a percentage of control cell numbers at matching time points (log y-axis, from 100% to 0.1% of control cell numbers). (*, $P < .05$, **, $P < .01$ compared to unlabeled girentuximab, two-way analysis of variance with Bonferroni's multiple comparison test, n = 3 cultures/group).

time, as confirmed with macroscopic examination of metastatic deposits in the liver of control mice (Figure 5C). Quantification of BLI data indicated that, despite the variability of tumor burden values within the control population, a >90% mean reduction in hepatic tumor burden was observed in the mice treated with [^{177}Lu]Lu-girentuximab by Day 23 (radiance = 0.132 ± 0.063 of Day 0 values for matching mice in [^{177}Lu]Lu-girentuximab-treated animals, vs 12.98 ± 8.45 fold increase compared to Day 0 in control mice at Day 23, $P = .015$, paired t-test, n = 5 mice/group), when the first control mice reached ethical end point (Figure 5D). Together these results suggest that [^{177}Lu]Lu-girentuximab provides significant therapeutic benefit in CA-IX-dependent preclinical models of mCRC.

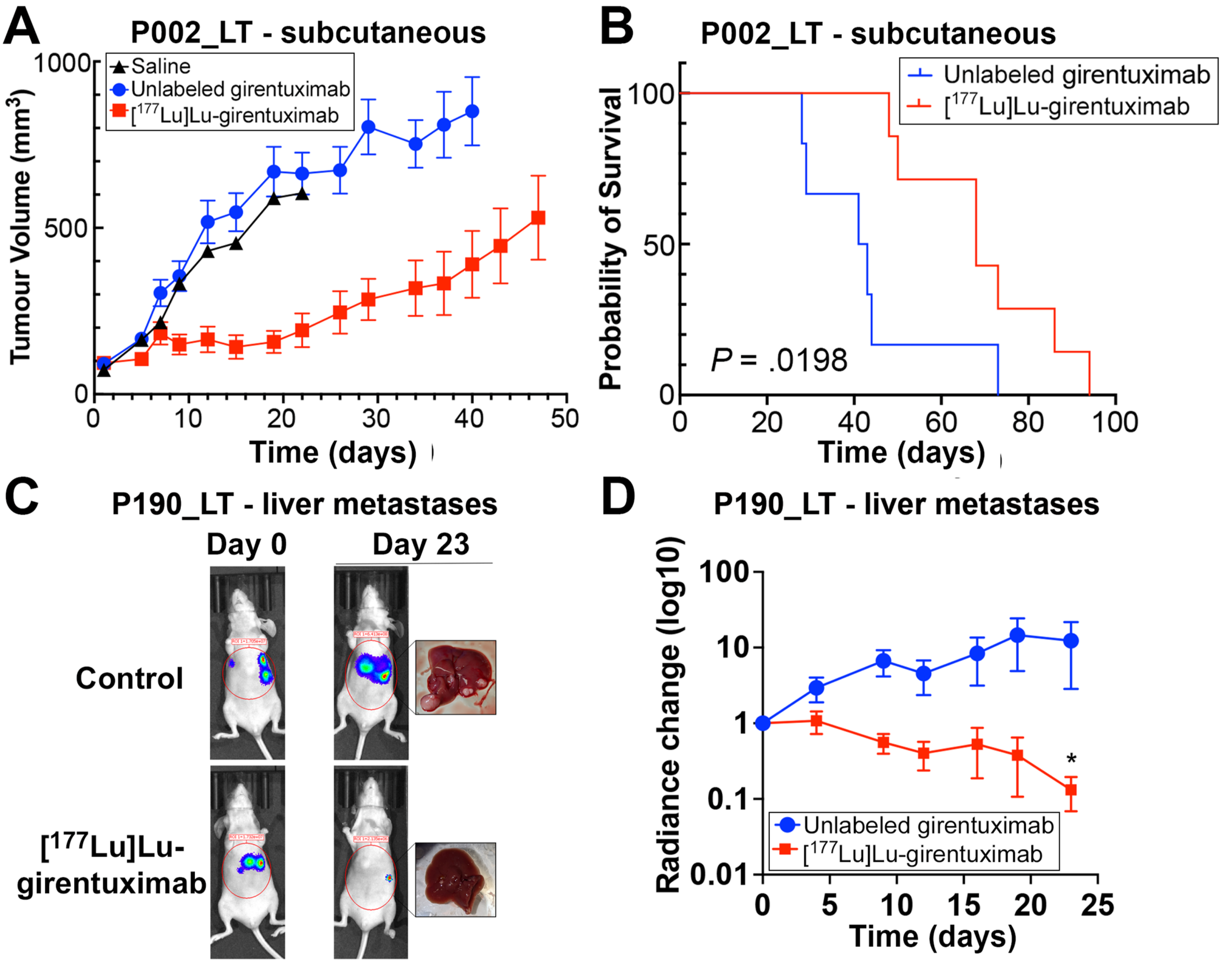

Figure 5. (A and B) Tumor volume (A) and probability of survival (B) in BALB/c nude mice bearing subcutaneous xenografts of P002_LT organoids, following a single-dose treatment with saline (back triangle), unlabeled girentuximab (blue dots) or [^{177}Lu]Lu-girentuximab (red squares) (*, $P = .0198$, Log-rank (Mantel-Cox) test, n = 7 mice/group). At Day 22 (the last measurement when all mice remained in the study), tumor growth inhibition was 82% of the unlabeled girentuximab group in [^{177}Lu]Lu-girentuximab-treated mice, $P < .0001$, unpaired T test of tumor growth between Day 1 and Day 22. (C and D) Representative bioluminescence imaging at Day 0 and Day 23 following treatment (C) and quantification of radiance variation across 23 days (D) in BALB/c nude mice bearing P190_LT organoid xenografts and treated once with unlabeled (blue dots) or [^{177}Lu]Lu-girentuximab (red squares) (*, $P = .0154$, paired *t*-test, n = 5 mice/group).

Discussion

CRC accounts for about 10% of global cancer incidence and mortality,[25] with nearly 50% of patients developing metastatic disease. Liver metastases are particularly common and are associated with poor prognosis, and 5-year survival is only around 13% for patients with metastatic CRC.[3] Despite advances in systemic chemotherapy, treatment remains limited by high toxicity, poor efficacy, and frequent relapse. Small and/or metabolically inactive liver metastases are often difficult to detect using standard imaging modalities,[26] increasing the risk of incomplete surgical resection. In addition, systemic chemotherapeutic regimens administered to patients with metastatic CRC exhibit high toxicity and are poorly efficient, with tumors frequently recurring after treatment.[3] The lack of reliable therapeutic targets and predictive biomarkers complicates patient stratification, treatment response monitoring and timely detection of recurrence. As shown in other cancers,[27] improvements in tumor-specific imaging and targeted therapeutic strategies would most likely enhance the survival outlook of patients with metastatic CRC.

Here, we demonstrated that CA-IX is a promising candidate for such strategies in CRC liver metastases. CA-IX is known to be strongly expressed in CRC primary tumors.[28,29] In this study, CA-IX protein expression was detected in 87% (40/46) of patient liver metastasis samples tested, with nearly half (21/46) displaying expression in over 50% of the tissue. The extent of CA-IX expression was consistent across chemo-naïve and neoadjuvant

chemotherapy-treated mCRC samples, suggesting that detection and therapeutic targeting of this protein may have broad applicability. The low expression of CA-IX in healthy liver tissue, it extracellular accessibility and enhanced target expression in hypoxic regions (this and previous work[30,31]), areas typically resistant to therapy, make it an attractive candidate for both imaging and therapeutic targeting. These results align with earlier findings in a few patients with metastatic clear cell Renal Cell Carcinoma,[14,32] supporting the rationale for a CA-IX-specific immune based theragnostic approach in mCRC.

In the present work we used radiolabled girentuximab due to its proven ability to bind CA-IX with high affinity and selectivity in human trials,[33,34] its well-tolerated profile in humans[15,33] and its promising outlook for scouting and therapeutic modalities.[11,12,14]

To evaluate CA-IX as an imaging target, we developed [^{89}Zr]Zr-girentuximab using a novel chelation strategy. Traditional zirconium labeling has used a multi-step approach attaching the bacterial siderophore desferroximine B (DFO) by reaction of desferrioxamine-N-succinyltetrafluorophenol ester (DFO-N-SucTFP) with the antibody. Synthesis of DFO-N-SucTFP requires protection of the hydroxyamic acid functional groups present in DFO by coordination to iron (III), followed by its removal prior to conjugation. In contrast, we employed a simplified synthesis method using desferrioxamine squaramide ethyl ester (H_3DFOSq-OEt), which can be readily prepared in a one-pot reaction between DFO mesylate and diethylsquarate without the need for protection of the hydroxamate functional groups.[35,36] Using this approach yielded H_3DFOSq-girentuximab with an average antibody to DFO ratio of 1:4. H_3DFOSq-girentuximab was radiolabeled with zirconium 89 by reaction of [^{89}Zr]Zr^{IV}-Oxalate, generating stable [^{89}Zr]Zr-DFOSq-girentuximab with preserved immunoreactivity and a high radiochemical yield (87%).[37]

In preclinical mouse models, [^{89}Zr]Zr-girentuximab demonstrated superior tumor uptake, tumor-to-background ratios, and spatial resolution compared to [^{18}F]F-FDG PET, corroborating previous demonstration in Renal Carcinoma clinical trials.[33] [^{89}Zr]Zr is usually internalized and trapped within cells after binding to the extracellular membrane, ensuring robust tumor uptake, an advantage over other long-life positron emitters such as iodine-124 ([^{124}I]I).[38] Its long half-life ($t_{1/2=}$78.4 h) and short positron range make it well-suited for imaging with stable probes such as monoclonal antibodies, enabling excellent spatial resolution.[38] Indeed, [^{89}Zr]Zr-girentuximab enabled detection of small (<1 cm), low-metabolism lesions that are often missed by conventional PET imaging. These properties support its potential for clinical use in patient stratification, staging, and treatment monitoring, particularly since CA-IX expression in liver metastases does not appear to be down-regulated after exposure to neo-adjuvant chemotherapy, at a time when decreased tumor metabolism can lead to decreased [^{18}F]F-FDG PET sensitivity.[39]

Clinical trials using radiolabeled girentuximab have yielded promising results in patients with metastatic ccRCC.[14,15] To assess therapeutic efficacy in mCRC, we radiolabeled girentuximab with lutetium-177 ([^{177}Lu]Lu) and tested it in patient-derived organoids and xenograft models. A single dose of [^{177}Lu]Lu-girentuximab significantly reduced tumor growth in 2 of 6 organoid lines in vitro, corresponding to organoids exhibiting WT or single nucleotide variant mutations of TP53. In contrast, organoids carrying frameshift TP53 mutations (insertions or deletions) were unresponsive to treatment. While several *TP53* mutations have been shown to reduce radiosensitivity, the mutational landscape of *TP53* is extremely complex and there is no complete correlation between *TP53* gene status and response to radiation therapy.[40,41] Our results suggest that some metastatic tumors carrying somatic missense variants may remain sensitive to [^{177}Lu]Lu radiation. In vivo, single dose [^{177}Lu]Lu-girentuximab treatment led to a strong and significant reduction of tumor burden in both subcutaneous and orthotopic mouse xenografts. Despite prior reports suggesting that lysosomal entrapment and removal of the nuclides by liver hepatocytes and splenic macrophages[42] might reduce the efficacy of [^{177}Lu]Lu-conjugated antibodies, our results highlight the ability of [^{177}Lu]Lu-girentuximab to target metastatic tumors located in the liver, predominant site of metastasis in CRC patients. Coadministration of [^{89}Zr]Zr- and [^{177}Lu]Lu-girentuximab confirmed similar biodistribution and tumor uptake, validating the potential of [^{89}Zr]Zr-girentuximab as a diagnostic companion for therapeutic planning. Altogether these results highlight the potential of CA-IX–targeted radioimmunotherapy in CRC liver metastases.

Of note, although [^{177}Lu]Lu is well tolerated in many patients, myelotoxicity and thrombocytopenia induced by the β-emitting nuclides often limit multiple radio immunotherapy cycles in cancer.[14] Indeed a Phase 2 clinical study investigating the therapeutic potential of [^{177}Lu]Lu-girentuximab in advanced renal cell carcinoma reported significant thrombocytopenia, highlighting the need to optimize dosing.[14] While consensus guidelines on expected and acceptable toxicities have been developed for peptide-based [^{177}Lu]Lu therapies,[43,44] the toxicity profile of antibody-based [^{177}Lu]Lu is only now becoming clearer,[44,45] with some indication that fractionation may not confer significantly greater tolerability.[45] Current data suggests that dose-limiting toxicities are likely to match antibody clearance patterns, reducing renal and intenstinal radiation doses, while increasing the dose to liver and bone marrow.[43] Multicycle dosimetry studies of [^{177}Lu]Lu-girentuximab will therefore be necessary to better understand the safety profile of this approach and to identify dose-limiting toxicities to nontarget organs in patients with mCRC. Personalized dosimetry derived from quantitative PET imaging with the [^{89}Zr]Zr-girentuxumab may offer the best route to identify optimal patient-specific dosing regimen.

Conclusion

In conclusion, the present study provides strong evidence to support the potential clinical utility of radiolabeled

girentuximab in a subset of patients with mCRC. The maintained expression of CA-IX in chemo-treated metastases suggest that using [^{89}Zr]Zr-girentuximab imaging at the time of staging, followed by [^{177}Lu]Lu-girentuximab treatment in patients with CA-IX-high tumors, may be useful in unresectable patients who received neoadjuvant chemotherapy, as well as in patients who are unable to tolerate chemotherapy and currently have no treatment options. Indeed, considering the high chemotherapy-associated toxicity in patients with mCRC,[46] the absence of side effects of [^{177}Lu]Lu-girentuximab in the 2 mouse models used suggests it may provide a better efficacy/morbidity ratio in a subset of patients with metastatic CRC. Patients with *TP53* WT tumors would strongly benefit from this treatment, and our results suggest that some patients with TP53 single nucleotide variant may also show strong responses to [^{177}Lu]Lu-girentuximab. As shown in advanced clear cell renal cell carcinoma,[47] combining subtherapeutic doses of [^{177}Lu]Lu-girentuximab with immune checkpoint inhibitors may also enhance treatment response in metastatic CRC, particularly in patients with liver metastases that typically respond poorly to immunotherapy.[48,49] We expect that antibody-based therapies will benefit from imaging data in much the same way as peptide-based [^{177}Lu]Lu therapies, where treatment decisions are guided by theranostic imaging. Factors such as metastasis location as well as amount and homogeneity of tracer uptake in tumor lesions will inform patient selection, prognosis, and therapy optimization.[43]

Supplementary Materials

Material associated with this article can be found in the online version at https://doi.org/10.1016/j.gastha.2025.100871.

References

1. Xi Y, Xu P. Global colorectal cancer burden in 2020 and projections to 2040. Transl Oncol 2021;14:101174.
2. Pan Z, Peng J, Lin J, et al. Is there a survival benefit from adjuvant chemotherapy for patients with liver oligometastases from colorectal cancer after curative resection? Cancer Commun (Lond) 2018;38:29.
3. Biller LH, Schrag D. Diagnosis and treatment of metastatic colorectal cancer: a review. JAMA 2021;325:669–685.
4. Andre T, Shiu KK, Kim TW, et al. Pembrolizumab in microsatellite-instability-high advanced colorectal cancer. N Engl J Med 2020;383:2207–2218.
5. Pastorekova S, Gillies RJ. The role of carbonic anhydrase IX in cancer development: links to hypoxia, acidosis, and beyond. Cancer Metastasis Rev 2019;38:65–77.
6. Chen KT, Seimbille Y. New developments in carbonic anhydrase IX-targeted fluorescence and nuclear imaging agents. Int J Mol Sci 2022;23:6125.
7. Rotermund A, Brandt S, Staege MS, et al. Differential CMS-related expression of cell surface carbonic anhydrases IX and XII in colorectal cancer models-implications for therapy. Int J Mol Sci 2023;24:5797.
8. Morgan KA, Rudd SE, Noor A, et al. Theranostic nuclear medicine with gallium-68, lutetium-177, copper-64/67, actinium-225, and lead-212/203 radionuclides. Chem Rev 2023;123:12004–12035.
9. Verhoeff SR, van Es SC, Boon E, et al. Lesion detection by [(89)Zr]Zr-DFO-girentuximab and [(18)F]FDG-PET/CT in patients with newly diagnosed metastatic renal cell carcinoma. Eur J Nucl Med Mol Imaging 2019;46:1931–1939.
10. Roussel E, Capitanio U, Kutikov A, et al. Novel imaging methods for renal mass characterization: a collaborative review. Eur Urol 2022;81:476–488.
11. Stillebroer AB, Franssen GM, Mulders PF, et al. ImmunoPET imaging of renal cell carcinoma with (124)I- and (89)Zr-labeled anti-CAIX monoclonal antibody cG250 in mice. Cancer Biother Radiopharm 2013;28:510–515.
12. Cheal SM, Punzalan B, Doran MG, et al. Pairwise comparison of 89Zr- and 124I-labeled cG250 based on positron emission tomography imaging and nonlinear immunokinetic modeling: in vivo carbonic anhydrase IX receptor binding and internalization in mouse xenografts of clear-cell renal cell carcinoma. Eur J Nucl Med Mol Imaging 2014;41:985–994.
13. Basaco T, Pektor S, Bermudez JM, et al. Evaluation of radiolabeled girentuximab in vitro and in vivo. Pharmaceuticals (Basel) 2018;11:132.
14. Muselaers CH, Boers-Sonderen MJ, van Oostenbrugge TJ, et al. Phase 2 study of lutetium 177-Labeled anti-carbonic anhydrase IX monoclonal antibody girentuximab in patients with advanced renal cell carcinoma. Eur Urol 2016;69:767–770.
15. Stillebroer AB, Boerman OC, Desar IM, et al. Phase 1 radioimmunotherapy study with lutetium 177-labeled anti-carbonic anhydrase IX monoclonal antibody girentuximab in patients with advanced renal cell carcinoma. Eur Urol 2013;64:478–485.
16. Shembrey C, Smith J, Grandin M, et al. Longitudinal monitoring of intra-tumoral heterogeneity using optical barcoding of patient-derived colorectal tumor models. Cancers (Basel) 2022;14:581.
17. Qin Y, Shembrey C, Smith J, et al. Laminin 521 enhances self-renewal via STAT3 activation and promotes tumor progression in colorectal cancer. Cancer Lett 2020;476:161–169.
18. Paquet-Fifield S, Koh SL, Cheng L, et al. Tight junction protein claudin-2 promotes self-renewal of human colorectal cancer stem-like cells. Cancer Res 2018;78:2925–2938.
19. Behrenbruch C, Foroutan M, Lind P, et al. Targeting of TP53-independent cell cycle checkpoints overcomes FOLFOX resistance in Metastatic Colorectal Cancer. bioRxiv 2021. http://doi.org/10.1101/2021.02.04.429849.
20. Li Y, Wang H, Oosterwijk E, et al. Antibody-specific detection of CAIX in breast and prostate cancers. Biochem Biophys Res Commun 2009;386:488–492.
21. Li G, Passebosc-Faure K, Lambert C, et al. The expression of G250/mn/CA9 antigen by flow cytometry: its possible implication for detection of micrometastatic renal cancer cells. Clin Cancer Res 2001;7:89–92.

22. Hektoen HH, Ree AH, Redalen KR, et al. Sulfamate inhibitor S4 influences carbonic anhydrase IX ectodomain shedding in colorectal carcinoma cells. J Enzyme Inhib Med Chem 2016;31:779–786.
23. Abou DS, Ku T, Smith-Jones PM. In vivo biodistribution and accumulation of 89Zr in mice. Nucl Med Biol 2011;38:675–681.
24. Niu T, Fan M, Lin B, et al. Current clinical application of lutetium-177 in solid tumors (Review). Exp Ther Med 2024;27:225.
25. Morgan E, Arnold M, Gini A, et al. Global burden of colorectal cancer in 2020 and 2040: incidence and mortality estimates from GLOBOCAN. Gut 2023;72:338–344.
26. Borello A, Russolillo N, Lo Tesoriere R, et al. Diagnostic performance of the FDG-PET/CT in patients with resected mucinous colorectal liver metastases. Surgeon 2021;19:e140–e145.
27. Bodei L, Herrmann K, Schöder H, et al. Radiotheranostics in oncology: current challenges and emerging opportunities. Nat Rev Clin Oncol 2022;19:534–550.
28. Korkeila E, Talvinen K, Jaakkola PM, et al. Expression of carbonic anhydrase IX suggests poor outcome in rectal cancer. Br J Cancer 2009;100:874–880.
29. Tupá V, Drahošová S, Grendár M, et al. Expression and association of carbonic anhydrase IX and cyclooxygenase-2 in colorectal cancer. Pathol Res Pract 2019;215:705–711.
30. Takacova M, Holotnakova T, Barathova M, et al. Src induces expression of carbonic anhydrase IX via hypoxia-inducible factor 1. Oncol Rep 2010;23:869–874.
31. Wykoff CC, Beasley NJ, Watson PH, et al. Hypoxia-inducible expression of tumor-associated carbonic anhydrases. Cancer Res 2000;60:7075–7083.
32. Muselaers CH, Boerman OC, Oosterwijk E, et al. Indium-111-labeled girentuximab immunoSPECT as a diagnostic tool in clear cell renal cell carcinoma. Eur Urol 2013;63:1101–1106.
33. Merkx RIJ, Lobeek D, Konijnenberg M, et al. Phase I study to assess safety, biodistribution and radiation dosimetry for (89)Zr-girentuximab in patients with renal cell carcinoma. Eur J Nucl Med Mol Imaging 2021;48:3277–3285.
34. van Oostenbrugge T, Mulders P. Targeted PET/CT imaging for clear cell renal cell carcinoma with radiolabeled antibodies: recent developments using girentuximab. Curr Opin Urol 2021;31:249–254.
35. Rudd SE, Noor A, Morgan KA, et al. Diagnostic positron emission tomography imaging with Zirconium-89 desferrioxamine B squaramide: from bench to bedside. Acc Chem Res 2024;57:1421–1433.
36. Rudd SE, Roselt P, Cullinane C, et al. A desferrioxamine B squaramide ester for the incorporation of zirconium-89 into antibodies. Chem Commun (Camb) 2016;52:11889–11892.
37. Noor A, Roselt PD, McGowan ER, et al. Automated synthesis of [(89)Zr]ZrCl(4), [(89)Zr]ZrDFOSquaramide-bisPh(PSMA) and [(89)Zr]ZrDFOSquaramide-TATE. EJNMMI Radiopharm Chem 2024;9:39.
38. Yoon JK, Park BN, Ryu EK, et al. Current perspectives on (89)Zr-PET imaging. Int J Mol Sci 2020;21:4309.
39. Spatz J, Holl G, Sciuk J, et al. Neoadjuvant chemotherapy affects staging of colorectal liver metastasis–a comparison of PET, CT and intraoperative ultrasound. Int J Colorectal Dis 2011;26:165–171.
40. Kennedy MC, Lowe SW. Mutant p53: it's not all one and the same. Cell Death Differ 2022;29:983–987.
41. Lau MYH, Islam Khan MZ, Law HKW. Molecular mechanism of radioresponsiveness in colorectal cancer: a systematic review. Genes (Basel) 2024;15:1257.
42. Kogos B, Cohen-Gould L, Rodriguez-Rodriguez C, et al. Electron microscopy of antibody-conjugated, lutetium-177 lanthanide gold-coated nanoparticles: proof of concept of targeted loci-A potential theranostic agent. Aip Adv. 2021;11. https://doi.org/10.1063/5.0044515.
43. Kratochwil C, Fendler WP, Eiber M, et al. Joint EANM/SNMMI procedure guideline for the use of (177)Lu-labeled PSMA-targeted radioligand-therapy ((177)Lu-PSMA-RLT). Eur J Nucl Med Mol Imaging 2023;50:2830–2845.
44. Nguyen H, Hird K, Cardaci J, et al. Lutetium-177 labelled Anti-PSMA monoclonal antibody (Lu-TLX591) therapy for metastatic prostate cancer: treatment toxicity and outcomes. Mol Diagn Ther 2024;28:291–299.
45. Niaz MJ, Batra JS, Walsh RD, et al. Pilot study of hyperfractionated dosing of lutetium-177-Labeled antiprostate-specific membrane antigen monoclonal antibody J591 ((177) Lu-J591) for metastatic castration-resistant prostate cancer. Oncologist 2020;25:477-e895.
46. Han CJ, Ning X, Burd CE, et al. Chemotoxicity and associated risk factors in colorectal cancer: a systematic review and meta-analysis. Cancers (Basel) 2024;16:2597.
47. Kleinendorst SC, Oosterwijk E, Molkenboer-Kuenen J, et al. Towards effective CAIX-targeted radionuclide and checkpoint inhibition combination therapy for advanced clear cell renal cell carcinoma. Theranostics 2024;14:3693–3707.
48. Chen EX, Loree JM, Titmuss E, et al. Liver metastases and immune checkpoint inhibitor efficacy in patients with refractory metastatic colorectal cancer: a secondary analysis of a randomized clinical trial. JAMA Netw Open 2023;6:e2346094.
49. Fakih M, Raghav KPS, Chang DZ, et al. Regorafenib plus nivolumab in patients with mismatch repair-proficient/microsatellite stable metastatic colorectal cancer: a single-arm, open-label, multicentre phase 2 study. EClinicalMedicine 2023;58:101917.

Received March 5, 2025. Accepted December 19, 2025.

Correspondence:
Address correspondence to: Frédéric Hollande, PhD, Department of Clinical Pathology, Victorian Comprehensive Cancer Centre, The University of Melbourne, Level 10, 305 Grattan Street, Melbourne, VIC 3000, Australia. e-mail: frederic.hollande@unimelb.edu.au.

Acknowledgments:
The authors wish to acknowledge Michael Wheatcroft (Telix Pharmaceuticals) for his support throughout the project and for providing girentuximab for this work. They are also grateful to the Victorian Centre for Functional Genomics and the Molecular Genomics Core (Peter MacCallum Cancer Centre, Melbourne, Australia) for their technical expertise in support of this work, to Nick

Clemons for the generous gift of pFUGW/Luc2-GFP, as well as Sepideh Foroutan and Joep Vissers for help with TP53 gene mutation identification. The authors acknowledge the National Health and Medical Research Council (PSD). They acknowledge the Bio21 Mass Spectrometry and Proteomics Facility for instrumentation and technical support, and financial support from the Australian Cancer Research Foundation for the purchase of an NMR spectrometer. They thank the animal technicians of Models of Cancer Translational Research Centre (Peter MacCallum Cancer Centre, Melbourne, Australia) for their technical contributions to this work.

Authors' Contributions:

Funding acquisition: Frédéric Hollande, Alexander G. Heriot, Benjamin Blyth, Paul S. Donnelly, Peter D. Roselt, Benjamin Loveday; Design/conceptualization: Frédéric Hollande, Alexander G. Heriot, Benjamin Blyth, Paul S. Donnelly; Methodology: Dijina Swaroop, Jai Smith, Jessica Van Zuykelom, Asif Noor, Paul S. Donnelly, Frédéric Hollande; Data curation and/or analysis: Dijina Swaroop, Jai Smith, Jessica Van Zuykelom, Asif Noor, Robin A. Wagner, Tamara Vu, Kelly Waldeck, Benjamin Blyth; Investigation/experiments: Dijina Swaroop, Jai Smith, Jessica Van Zuykelom, Asif Noor, Sarah Lee, Robin A. Wagner, Kelly Waldeck; Visualization: Dijina Swaroop, Jai Smith, Jessica Van Zuykelom, Asif Noor, Robin A. Wagner, Sarah Lee, Kelly Waldeck, Frédéric Hollande; Resource providing: Alexander G. Heriot, Benjamin Loveday, Peter D. Roselt, Paul S. Donnelly; Manuscript writing: Dijina Swaroop, Jai Smith, Jessica Van Zuykelom, Asif Noor, Benjamin Blyth, Paul S. Donnelly, Frédéric Hollande.

Conflicts of Interest:

These authors disclose the following: Asif Noor and Paul S. Donnelly are listed as inventors on intellectual property broadly related to this area of research that has been licensed from the University of Melbourne to Telix Pharmaceuticals. Paul S. Donnelly has potential financial interests in Clarity Pharmaceuticals and Telix Pharmaceuticals. Unrelated to this research, the University of Melbourne (Paul S. Donnelly) has received research funding from Telix Pharmaceuticals. The remaining authors disclose no conflicts.

Funding:

This work was supported by a Priority-driven Collaborative Cancer Research Scheme grant from Cancer Australia (Grant #1184859) and a project grant from the National Health and Medical Research Council of Australia (GNT1164081) to Frederic Hollande.

Ethical Statement:

Informed consent was provided by each patient, and the use of patient tissues was approved by the Peter MacCallum Cancer Centre Human Research Ethics Committee (HREC/15/PMCC/112) and the Melbourne Health Human Research Ethics Committee (HREC/61352/MH-2020). Animal experiments were performed under approval from the Peter MacCallum Animal Ethics Committee (agreements E562 and E654) and in accordance with the Australian Code for the Care and Use of Animals for Scientific Purposes.

Data Transparency Statement:

cG250 (girentuximab) was provided by Telix Pharmaceuticals. Other study materials and protocols will be made available upon request.

Reporting Guidelines:

Reporting Guidelines were not applicable for this article type.

Gastro Hep Advances, Volume 5

Supplemental information

Carbonic Anhydrase-IX Is a Specific and Sensitive Theragnostic Target for Imaging and Radioimmunotherapy in Metastatic Colorectal Cancer

Dijina Swaroop, Jai Smith, Jessica Van Zuykelom, Asif Noor, Robin A. Wagner, Tamara Vu, Sarah Lee, Alexander G. Heriot, Benjamin Loveday, Kelly Waldeck, Peter D. Roselt, Benjamin Blyth, Paul S. Donnelly, and Frédéric Hollande

SUPPLEMENTAL METHODS

Organoid culture

Organoids were cultured in 24 well-cell culture plates in a growth factor reduced Matrigel matrix (Corning, 356231). The organoids were maintained in Advanced DMEM/F12 (2634010, ThermoFisher Scientific) or HPLM (#A4[89]9101, ThermoFisher Scientific) culture medium for *in vitro* and *in vivo* experiments. These media were supplemented with Glutamax (2 mM; Life Technologies, #35050-061), penicillin-streptomycin (10 U/mL; Life Technologies, #15140-122), recombinant human EGF (50 ng/mL, Miltenyi Biotec, 130-097-749), human Gastrin I (1 µg/mL, Merck, G9145), N-acetyl cysteine (1 mM, Merck, A9165), B27 (2X; Life Technologies, 17504044), A83-01 (ALK inhibitor; 500 nM, Merck, SML0788), SB202190 (p38/MAPK inhibitor; 10 nM, Miltenyi Biotec, 130-106-275) and YP-27632 (ROCK inhibitor; 10 µM, Abcam, 120129) at 37 ˚C in a humidified atmosphere with 5% CO2. Organoids were passaged at a split ratio of 1:3–1:6 every 3-7 days at 70%-80% confluency.

Immunohistochemistry

FFPE sections were deparaffinised in xylene solution and rehydrated in ethanol. The antigens were retrieved from the sample using the pressure cooker method by heating in a 10mM citrate buffer solution (pH = 6) at 125 °C for 15 min. Subsequently, the endogenous peroxidase activity was blocked with H_2O_2 solution for 1 minute. The samples were blocked in 2% horse serum, followed by incubation in primary antibody for 30 min at room temperature. The samples were rinsed in PBS and incubated with the secondary antibody for 15 min.

The tissue sections were stained by adding 100µl DAB substrate solution (05% DAB - 0.015% H_2O_2 in PBS) and incubated for <5 min until the desired colour intensity was reached. The slides were subsequently rinsed with PBS and counter-stained by immersing sides in Hematoxylin for 1-2 min.

For histological analysis of organoids, Matrigel domes containing organoids were fixed with 10% neutral buffered formalin containing 0.5% glutaraldehyde for an hour and carefully dislodged using a P_{1000} pipette tip. The organoids were submerged in 80% ethanol for storage until used. The ethanol was discarded for further analysis, and the Matrigel domes were transferred to a cryomold containing 1% Agarose (in PBS). The cryomold was subsequently processed, as mentioned previously.

Western Blotting

Western blotting was mostly performed as described previously.[17] Whole cell lysates of the organoids were prepared using RIPA lysis buffer containing protease inhibitors. The sample protein concentration was determined by BCA assay (23252, Pierce™ Microplate BCA Protein Assay Kit). For western blotting, samples were subjected to 10% SDS-PAGE and transferred to PVDF membranes (IPVH00010, Immobilon®-P PVDF Membrane, Merck). The membranes were blocked with 5% skimmed milk and then incubated overnight in primary antibodies: CA-IX (NB100-417, Novus Biologicals) and β-actin (A3B53, Sigma). Subsequently, the

membranes were washed and incubated for an hour in the respective secondary antibody [(antimouse: Vector, ImmPRESS®-VR Horse Anti-Mouse IgG Polymer Reagent -30028) or (antirabbit: Vector, ImmPRESS®-VR Horse Anti-Rabbit IgG Polymer Reagent -30026)] for an hour at room temperature. The bands were visualised using ECL reagent (170-5061, Biorad)) and quantified with Image Lab software (BIORAD, version 6.1).

Girentuximab radiolabelling

Synthesis of DFOSq-girentuximab:

To a solution of girentuximab antibody (1 equiv., 1 mg, 166 μL) in borate buffer (pH 9.0, 0.1M, 784 μL) was added DFOSq (40 equiv., 185 μg in DMSO, 50 μL) at room temperature to give a final antibody concentration of 1 mg/mL of the reaction mixture. The reaction mixture was incubated at room temperature for 18 hours then filtered using Amicon® 100 kDa centrifugal filters. The crude product was washed on the filter with 0.9% saline and the concentrate was collected and diluted with 0.9% saline (250 μL) to give DFOSq-girentuximab (4 mg/mL). The product was analyzed by LC-MS which indicated a mixture of girentuximab with 1-6 chelators with an average 4.2 chelators/antibody.

Synthesis of DOTA-girentuximab:

To a solution of girentuximab antibody (1 equiv., 3 mg, 654 μL) was added $NaHCO_3$ solution (1 M, 73 μL) and DOTA-NHS (10 equiv., 155 μg in DMSO, 38 μL) at room temperature to give a final antibody concentration of 4 mg/mL of the reaction mixture. The reaction mixture was incubated at room temperature for 18 hours then filtered using Amicon® 100 kDa centrifugal filters. The crude product was washed on the filter with 0.9% saline and the concentrate was collected and diluted with 0.9% saline (400 μL) to give DFOSq-girentuximab (7.5 mg/mL). The product was analyzed by LC-MS which indicated a mixture of girentuximab with 1-6 chelators with an average 3.8 chelators/antibody.

Animal Experiments

Subcutaneous inoculation: For subcutaneous inoculation, P002_LT organoids were injected subcutaneously into the right flanks of the mice with a 1:1 mixture of Matrigel and PBS in a final volume of 50 μL/mouse. Mice were weighed and tumour growth was monitored twice weekly using electronic calipers.

Intrasplenic inoculation: Mice (7–14-week-old) mice were intraperitoneally injected with an anaesthetic buprenorphine solution. A skin and peritoneal wall incision was performed to locate the spleen under the rib cage on the left side of the mouse's abdomen. The spleen was gently exteriorised from the abdominal cavity, and Luciferase 2-expressing P190_A_LT organoids were injected into the spleen extremity in 20uL of 1:1 Matrigel/PBS mix. After 2 minutes, a total splenectomy was performed by ligating the blood vessels with wound clips and suturing the peritoneum and the skin. Liver metastasis volume was monitored via bioluminescence imaging, whereby mice bearing xenografts were intraperitoneally administered with D-

luciferin (150mg/kg body weight). 20 minutes after injection, mice were anaesthetised using 2% isoflurane mixed with oxygen and imaged using an IVIS Lumina II (Perkin Elmer) imaging system, as per our previous work [19].

Upon showing signs of distress or when mice reached experimental endpoint, they were euthanised by asphyxiation with C02 and their subcutaneous or liver tumours, respectively, were excised and fixed in 10% neutral buffered formalin.

SUPPLEMENTAL METHODS

Organoid culture

Organoids were cultured in 24 well-cell culture plates in a growth factor reduced Matrigel matrix (Corning, 356231). The organoids were maintained in Advanced DMEM/F12 (2634010, ThermoFisher Scientific) or HPLM (#A4^{89}9101, ThermoFisher Scientific) culture medium for *in vitro* and *in vivo* experiments. These media were supplemented with Glutamax (2 mM; Life Technologies, #35050-061), penicillin-streptomycin (10 U/mL; Life Technologies, #15140-122), recombinant human EGF (50 ng/mL, Miltenyi Biotec, 130-097-749), human Gastrin I (1 µg/mL, Merck, G9145), N-acetyl cysteine (1 mM, Merck, A9165), B27 (2X; Life Technologies, 17504044), A83-01 (ALK inhibitor; 500 nM, Merck, SML0788), SB202190 (p38/MAPK inhibitor; 10 nM, Miltenyi Biotec, 130-106-275) and YP-27632 (ROCK inhibitor; 10 µM, Abcam, 120129) at 37 ˚C in a humidified atmosphere with 5% CO2. Organoids were passaged at a split ratio of 1:3–1:6 every 3-7 days at 70%-80% confluency.

Immunohistochemistry

FFPE sections were deparaffinised in xylene solution and rehydrated in ethanol. The antigens were retrieved from the sample using the pressure cooker method by heating in a 10mM citrate buffer solution (pH = 6) at 125 °C for 15 min. Subsequently, the endogenous peroxidase activity was blocked with H_2O_2 solution for 1 minute. The samples were blocked in 2% horse serum, followed by incubation in primary antibody for 30 min at room temperature. The samples were rinsed in PBS and incubated with the secondary antibody for 15 min.

The tissue sections were stained by adding 100µl DAB substrate solution (05% DAB - 0.015% H_2O_2 in PBS) and incubated for <5 min until the desired colour intensity was reached. The slides were subsequently rinsed with PBS and counter-stained by immersing sides in Hematoxylin for 1-2 min.

For histological analysis of organoids, Matrigel domes containing organoids were fixed with 10% neutral buffered formalin containing 0.5% glutaraldehyde for an hour and carefully dislodged using a P_{1000} pipette tip. The organoids were submerged in 80% ethanol for storage until used. The ethanol was discarded for further analysis, and the Matrigel domes were transferred to a cryomold containing 1% Agarose (in PBS). The cryomold was subsequently processed, as mentioned previously.

Western Blotting

Western blotting was mostly performed as described previously.[17] Whole cell lysates of the organoids were prepared using RIPA lysis buffer containing protease inhibitors. The sample protein concentration was determined by BCA assay (23252, Pierce™ Microplate BCA Protein Assay Kit). For western blotting, samples were subjected to 10% SDS-PAGE and transferred to PVDF membranes (IPVH00010, Immobilon®-P PVDF Membrane, Merck). The membranes were blocked with 5% skimmed milk and then incubated overnight in primary antibodies: CA-IX (NB100-417, Novus Biologicals) and β-actin (A3B53, Sigma). Subsequently, the

membranes were washed and incubated for an hour in the respective secondary antibody [(antimouse: Vector, ImmPRESS®-VR Horse Anti-Mouse IgG Polymer Reagent -30028) or (antirabbit: Vector, ImmPRESS®-VR Horse Anti-Rabbit IgG Polymer Reagent -30026)] for an hour at room temperature. The bands were visualised using ECL reagent (170-5061, Biorad)) and quantified with Image Lab software (BIORAD, version 6.1).

Girentuximab radiolabelling

Synthesis of DFOSq-girentuximab:

To a solution of girentuximab antibody (1 equiv., 1 mg, 166 μL) in borate buffer (pH 9.0, 0.1M, 784 μL) was added DFOSq (40 equiv., 185 μg in DMSO, 50 μL) at room temperature to give a final antibody concentration of 1 mg/mL of the reaction mixture. The reaction mixture was incubated at room temperature for 18 hours then filtered using Amicon® 100 kDa centrifugal filters. The crude product was washed on the filter with 0.9% saline and the concentrate was collected and diluted with 0.9% saline (250 μL) to give DFOSq-girentuximab (4 mg/mL). The product was analyzed by LC-MS which indicated a mixture of girentuximab with 1-6 chelators with an average 4.2 chelators/antibody.

Synthesis of DOTA-girentuximab:

To a solution of girentuximab antibody (1 equiv., 3 mg, 654 μL) was added $NaHCO_3$ solution (1 M, 73 μL) and DOTA-NHS (10 equiv., 155 μg in DMSO, 38 μL) at room temperature to give a final antibody concentration of 4 mg/mL of the reaction mixture. The reaction mixture was incubated at room temperature for 18 hours then filtered using Amicon® 100 kDa centrifugal filters. The crude product was washed on the filter with 0.9% saline and the concentrate was collected and diluted with 0.9% saline (400 μL) to give DFOSq-girentuximab (7.5 mg/mL). The product was analyzed by LC-MS which indicated a mixture of girentuximab with 1-6 chelators with an average 3.8 chelators/antibody.

Animal Experiments

Subcutaneous inoculation: For subcutaneous inoculation, P002_LT organoids were injected subcutaneously into the right flanks of the mice with a 1:1 mixture of Matrigel and PBS in a final volume of 50 μL/mouse. Mice were weighed and tumour growth was monitored twice weekly using electronic calipers.

Intrasplenic inoculation: Mice (7–14-week-old) mice were intraperitoneally injected with an anaesthetic buprenorphine solution. A skin and peritoneal wall incision was performed to locate the spleen under the rib cage on the left side of the mouse's abdomen. The spleen was gently exteriorised from the abdominal cavity, and Luciferase 2-expressing P190_A_LT organoids were injected into the spleen extremity in 20uL of 1:1 Matrigel/PBS mix. After 2 minutes, a total splenectomy was performed by ligating the blood vessels with wound clips and suturing the peritoneum and the skin. Liver metastasis volume was monitored via bioluminescence imaging, whereby mice bearing xenografts were intraperitoneally administered with D-

luciferin (150mg/kg body weight). 20 minutes after injection, mice were anaesthetised using 2% isoflurane mixed with oxygen and imaged using an IVIS Lumina II (Perkin Elmer) imaging system, as per our previous work [19].

Upon showing signs of distress or when mice reached experimental endpoint, they were euthanised by asphyxiation with C02 and their subcutaneous or liver tumours, respectively, were excised and fixed in 10% neutral buffered formalin.
