# Supplementary material for: Carbonic Anhydrase-IX Is a Specific and Sensitive Theragnostic Target for Imaging and Radioimmunotherapy in Metastatic Colorectal Cancer
Source: Gastro Hep Adv. 2025 Dec 26;5(3):100871. doi: 10.1016/j.gastha.2025.100871 (PMC12860988; doi:10.1016/j.gastha.2025.100871)
Supplement: Tables A1–A4 and Figure A1 Legends [file mmc2.docx]

**Swaroop, Smith et al, 2024 Supplemental Figure and supplemental Table Legends**

**Supplemental Figure S1**

**(A)** Representative images reflecting the extracellular membrane detection of CA-IX in tumor xenografts generated from P002_LT and P295_LT organoid, respectively using immunohistochemistry (top panel) and immunofluorescent staining (bottom panel). **(B)** Similar distribution pattern of CA-IX mRNA expression in liver metastasis samples collected prior to adjuvant chemotherapy (chemo-naïve tissue, n = 32, left panel) or after neo-adjuvant chemotherapy exposure (n = 25, right panel); **(C)** Correlation between CA-IX immunohistochemistry scoring (as shown in figure 1 A-B) and CA-IX mRNA expression in matching metastasis samples (Spearman correlation, R = 0.73, *P* = .0002, n = 21 patients). **(D)** CA-IX mRNA expression profile across all metastasis samples (n = 57) and PDTOs (n = 15). **(E)** Representative example of H&E staining, hypoxic region staining (Hypoxiprobe, Pimonidazole Hydrochloride) and CA-IX immunostaining in a liver metastatic xenograft generated from P190_LT organoids. (Scale bars = 1mm (top panels) and 200μm (bottom)).

**Supplemental Table I**

Detailed Staining intensity and staining coverage scores, as well as resulting total IHC scores, following immunohistochemical staining of 46 liver metastasis samples. Indication of whether each sample was collected prior to chemotherapy or from a chemo-treated patient is also included.

**Supplemental Table II**

Mean biodistribution and standard deviation values of each organ collected 24, 48 or 144h after injection of [^89^Zr]Zr- or [^177^Lu]Lu-girentuximab, as shown in Figure 2B, C and D. n=5 mice per group unless otherwise indicated.

**Supplemental Table III**

Maximum Standardized Uptake Values (SUVmax) of tumors, and mean SUV ratios of tumor to background, liver and bone, with standard deviation, in P002_LT xenografts (as per Figure 2E). These values are calculated from PET imaging of mice 1h after injection of [^18^F]F-FDG or 24, 48 or 144h after injection of [^89^Zr]Zr-girentuximab (n = 5 mice per group). A representative example of this imaging is shown in Figure 3. Concomitant corroboration was obtained in 2 mice xenografted with P190_LT organoids.

**Supplemental Table IV**

*TP53* gene mutation status in the six patient-derived organoid lines used in this study, summarizing the mutation consequence, exon location, alteration of DNA and protein sequences (using the Human Genome Variation Society – HGVS – standard nomenclature), and variant class.
