# Supplementary material for: Carbonic Anhydrase-IX Is a Specific and Sensitive Theragnostic Target for Imaging and Radioimmunotherapy in Metastatic Colorectal Cancer
Source: Gastro Hep Adv. 2025 Dec 26;5(3):100871. doi: 10.1016/j.gastha.2025.100871 (PMC12860988; doi:10.1016/j.gastha.2025.100871)
Supplement: Extended PDF [file mmc7.pdf]

SUPPLEMENTAL METHODS

Organoid culture

Organoids were cultured in 24 well-cell culture plates in a growth factor reduced Matrigel matrix (Corning, 356231). The organoids were maintained in Advanced DMEM/F12 (2634010, ThermoFisher Scientific) or HPLM (#A4[89]9101, ThermoFisher Scientific) culture medium for *in vitro* and *in vivo* experiments. These media were supplemented with Glutamax (2 mM; Life Technologies, #35050-061), penicillin-streptomycin (10 U/mL; Life Technologies, #15140-122), recombinant human EGF (50 ng/mL, Miltenyi Biotec, 130-097-749), human Gastrin I (1 µg/mL, Merck, G9145), N-acetyl cysteine (1 mM, Merck, A9165), B27 (2X; Life Technologies, 17504044), A83-01 (ALK inhibitor; 500 nM, Merck, SML0788), SB202190 (p38/MAPK inhibitor; 10 nM, Miltenyi Biotec, 130-106-275) and YP-27632 (ROCK inhibitor; 10 µM, Abcam, 120129) at 37 ˚C in a humidified atmosphere with 5% CO2. Organoids were passaged at a split ratio of 1:3–1:6 every 3-7 days at 70%-80% confluency.

Immunohistochemistry

FFPE sections were deparaffinised in xylene solution and rehydrated in ethanol. The antigens were retrieved from the sample using the pressure cooker method by heating in a 10mM citrate buffer solution (pH = 6) at 125 °C for 15 min. Subsequently, the endogenous peroxidase activity was blocked with H_2O_2 solution for 1 minute. The samples were blocked in 2% horse serum, followed by incubation in primary antibody for 30 min at room temperature. The samples were rinsed in PBS and incubated with the secondary antibody for 15 min.

The tissue sections were stained by adding 100µl DAB substrate solution (05% DAB - 0.015% H_2O_2 in PBS) and incubated for <5 min until the desired colour intensity was reached. The slides were subsequently rinsed with PBS and counter-stained by immersing sides in Hematoxylin for 1-2 min.

For histological analysis of organoids, Matrigel domes containing organoids were fixed with 10% neutral buffered formalin containing 0.5% glutaraldehyde for an hour and carefully dislodged using a P_{1000} pipette tip. The organoids were submerged in 80% ethanol for storage until used. The ethanol was discarded for further analysis, and the Matrigel domes were transferred to a cryomold containing 1% Agarose (in PBS). The cryomold was subsequently processed, as mentioned previously.

Western Blotting

Western blotting was mostly performed as described previously.[17] Whole cell lysates of the organoids were prepared using RIPA lysis buffer containing protease inhibitors. The sample protein concentration was determined by BCA assay (23252, Pierce™ Microplate BCA Protein Assay Kit). For western blotting, samples were subjected to 10% SDS-PAGE and transferred to PVDF membranes (IPVH00010, Immobilon®-P PVDF Membrane, Merck). The membranes were blocked with 5% skimmed milk and then incubated overnight in primary antibodies: CA-IX (NB100-417, Novus Biologicals) and β-actin (A3B53, Sigma). Subsequently, the

membranes were washed and incubated for an hour in the respective secondary antibody [(antimouse: Vector, ImmPRESS®-VR Horse Anti-Mouse IgG Polymer Reagent -30028) or (antirabbit: Vector, ImmPRESS®-VR Horse Anti-Rabbit IgG Polymer Reagent -30026)] for an hour at room temperature. The bands were visualised using ECL reagent (170-5061, Biorad)) and quantified with Image Lab software (BIORAD, version 6.1).

Girentuximab radiolabelling

Synthesis of DFOSq-girentuximab:

To a solution of girentuximab antibody (1 equiv., 1 mg, 166 μL) in borate buffer (pH 9.0, 0.1M, 784 μL) was added DFOSq (40 equiv., 185 μg in DMSO, 50 μL) at room temperature to give a final antibody concentration of 1 mg/mL of the reaction mixture. The reaction mixture was incubated at room temperature for 18 hours then filtered using Amicon® 100 kDa centrifugal filters. The crude product was washed on the filter with 0.9% saline and the concentrate was collected and diluted with 0.9% saline (250 μL) to give DFOSq-girentuximab (4 mg/mL). The product was analyzed by LC-MS which indicated a mixture of girentuximab with 1-6 chelators with an average 4.2 chelators/antibody.

Synthesis of DOTA-girentuximab:

To a solution of girentuximab antibody (1 equiv., 3 mg, 654 μL) was added $NaHCO_3$ solution (1 M, 73 μL) and DOTA-NHS (10 equiv., 155 μg in DMSO, 38 μL) at room temperature to give a final antibody concentration of 4 mg/mL of the reaction mixture. The reaction mixture was incubated at room temperature for 18 hours then filtered using Amicon® 100 kDa centrifugal filters. The crude product was washed on the filter with 0.9% saline and the concentrate was collected and diluted with 0.9% saline (400 μL) to give DFOSq-girentuximab (7.5 mg/mL). The product was analyzed by LC-MS which indicated a mixture of girentuximab with 1-6 chelators with an average 3.8 chelators/antibody.

Animal Experiments

Subcutaneous inoculation: For subcutaneous inoculation, P002_LT organoids were injected subcutaneously into the right flanks of the mice with a 1:1 mixture of Matrigel and PBS in a final volume of 50 μL/mouse. Mice were weighed and tumour growth was monitored twice weekly using electronic calipers.

Intrasplenic inoculation: Mice (7–14-week-old) mice were intraperitoneally injected with an anaesthetic buprenorphine solution. A skin and peritoneal wall incision was performed to locate the spleen under the rib cage on the left side of the mouse's abdomen. The spleen was gently exteriorised from the abdominal cavity, and Luciferase 2-expressing P190_A_LT organoids were injected into the spleen extremity in 20uL of 1:1 Matrigel/PBS mix. After 2 minutes, a total splenectomy was performed by ligating the blood vessels with wound clips and suturing the peritoneum and the skin. Liver metastasis volume was monitored via bioluminescence imaging, whereby mice bearing xenografts were intraperitoneally administered with D-

luciferin (150mg/kg body weight). 20 minutes after injection, mice were anaesthetised using 2% isoflurane mixed with oxygen and imaged using an IVIS Lumina II (Perkin Elmer) imaging system, as per our previous work [19].

Upon showing signs of distress or when mice reached experimental endpoint, they were euthanised by asphyxiation with C02 and their subcutaneous or liver tumours, respectively, were excised and fixed in 10% neutral buffered formalin.
